# Graves’ ophthalmopathy: low-dose dexamethasone reduces retinoic acid receptor-alpha gene expression in orbital fibroblasts

**DOI:** 10.20945/2359-3997000000044

**Published:** 2018-05-07

**Authors:** Sarah Santiloni Cury, Miriane Oliveira, Maria Teresa Síbio, Sueli Clara, Renata De Azevedo Melo Luvizotto, Sandro Conde, Edson Nacib Jorge, Vania dos Santos Nunes, Célia Regina Nogueira, Gláucia Maria Ferreira da Silva Mazeto

**Affiliations:** 1 Universidade Estadual Paulista Universidade Estadual de São Paulo Faculdade de Medicina de Botucatu Departamento de Medicina Interna, Divisão de Endocrinologia Botucatu SP Brasil Divisão de Endocrinologia, Departamento de Medicina Interna, Faculdade de Medicina de Botucatu, Universidade Estadual de São Paulo (Unesp), Botucatu, SP, Brasil; 2 Instituto Federal de Educação, Ciência e Tecnologia de São Paulo Instituto Federal de Educação, Ciência e Tecnologia do Estado de São Paulo São Roque SP Brasil Instituto Federal de Educação, Ciência e Tecnologia do Estado de São Paulo (IFSP), São Roque, SP, Brasil; 3 Universidade Estadual Paulista Universidade Estadual de São Paulo Faculdade de Medicina de Botucatu Departamento de Oftalmologia, Otorrinolaringologia e Cirurgia de Cabeça e Pescoço Botucatu SP Brasil Departamento de Oftalmologia, Otorrinolaringologia e Cirurgia de Cabeça e Pescoço, Faculdade de Medicina de Botucatu, Universidade Estadual de São Paulo (Unesp), Botucatu, SP, Brasil

**Keywords:** Receptors, retinoic acid, gene expression, graves ophthalmopathy

## Abstract

**Objective:**

Graves’ ophthalmopathy (GO) is an autoimmune disease that leads to ocular proptosis caused by fat accumulation and inflammation, and the main treatment is corticosteroid therapy. Retinoid acid receptor-alpha (RARα) seems to be associated with inflammation and adipocyte differentiation. This study aimed to assess the effect of glucocorticoid treatment on orbital fibroblasts of GO patient treated or not with different glucocorticoid doses.

**Materials and methods:**

Orbital fibroblasts collected during orbital decompression of a female patient with moderately severe/severe GO were cultivated and treated with 10 nM and 100 nM dexamethasone (Dex). *rRAR*α gene expression in the treated and untreated cells was then compared.

**Results:**

Fibroblast *RAR*α expression was not affected by 100 nM Dex. On the other hand, *RAR*α expression was 24% lower in cells treated with 10 nM Dex (p < 0.05).

**Conclusions:**

Orbital fibroblasts from a GO patient expressed the *RAR*α gene, which was unaffected by higher, but decreased with lower doses of glucocorticoid.

## INTRODUCTION

Graves’ ophthalmopathy (GO) is characterized by expansion of the orbital tissue due to immune-mediated inflammation and adipocyte proliferation secondary to orbital fibroblast (OF) differentiation (1). The main treatment for moderate to severe cases is corticosteroid therapy, which carries some risks (2,3), while other therapeutic approaches have provided with limited results in a reasonable number of patients (4). Thus, new GO treatment options are being sought.

Retinoic acid (RA) is the biologically active form of vitamin A. It is widely used in clinical practice, especially in the form of isotretinoin for the treatment of acne (5). Nonetheless, studies indicate that RA may also play a role in adipogenesis (6,7) and inflammatory/autoimmune processes (8). The effects of RA depend on nuclear receptors from two families of nuclear transcription regulators: RA receptor (RAR) and retinoid X receptor (RXR) (9,10). RAR, but not RXR, has affinity for all forms of RA (11). RARα stands out as it is the most frequently expressed RAR in cells (12).

The OF of GO patients express RA receptors, including RARα, and retinoids could inhibit adipocyte growth and differentiation, and induce apoptosis in these cells, consequently presenting therapeutic potential in GO (13,14). However, the development of severe GO has been associated with the simultaneous use of RA, recombinant thyroid-stimulating hormone (TSH), and radioactive iodine (15). Hence, the use of RA in GO may or may not have satisfactory results, depending on patient clinical condition, associated treatments, and RAR existence/functionality/response. Other studies on the use of RA or the expression of its nuclear receptors in GO have not been found.

We recently reported the case of a 62-year-old female patient with inactive, moderately severe to severe GO, not previously treated with glucocorticoid, whose fibroblasts expressed the peroxisome proliferator-activated receptor-gamma (PPAR_γ_) and estrogen receptor-alpha (ERα) genes. Interestingly, this gene expression responded to glucocorticoids in a dose-related manner (16,17). Thus, the aim of this study was to evaluate the *RAR*α gene expression by cultured OF from GO patient, which were treated or not with different glucocorticoid doses.

## MATERIALS AND METHODS

The present study, approved by the Research Ethics Committee of Botucatu Medical School under process number 4037-2011), assessed *RAR*α gene expression in cultured OF treated or not with 10 nM or 100 nM dexamethasone (Dex). The cells were initially obtained during orbital decompression performed at the Clinics Hospital (HC) of the Botucatu Medical School (FMB) – Unesp, placed in a falcon tube containing the medium 199 (LCG^®^) and antibiotic, and transported to the experimental laboratory of medical clinic, where the cells were cultivated, as previously described (16,17). Once the fibroblasts in the wells reached 80% confluence, they were treated with 10 nM or 100 nM Dex in biological triplicates, and their *RAR*α gene expression was compared with that of untreated fibroblasts (control group, C) (16,17). After treatment, the culture medium 199 was removed, and cells were collected from the plates with 400 µL of TRIzol^®^ (Life Tech, USA) for RNA extraction, also perfomed with TRIzol^®^. Sample integrity was verified by running them for 30 minutes in 1% agarose gel at a voltage of 80 mV. The RNA samples were then analyzed by spectrophotometry. Samples with 260:280 ratios below 1.6 were discarded because of protein contamination. Complementary deoxyribonucleic acid (cDNA) was synthesized from 1 µg of RNA by reverse transcription using the High-capacity cDNA Reverse Transcription Kit (Applied Biosystems, CA, USA). In short, the following were added to the RNA sample: 2 µL of 10x buffer, 0.8 µL of 100 mM dNTP mix, 2 µL of random primer, 1 µL of RNase inhibitor, 1 µL of reverse transcriptase, and 12.2 µL of nuclease-free water. Samples were incubated at 25ºC for 10 minutes, at 37ºC for 120 minutes, at 85ºC for 5 seconds, and maintained at 4ºC. Each cDNA sample was analyzed by TaqMan Assays (Life Technologies, USA) containing specific primers (ESR1: estrogen receptor alpha and cyclophilin: internal control) for the target RNAs. Each reaction used 10 μL of TaqMan^®^ Universal PCR Master Mix (Life Technologies, USA) and 3 μL of the reverse transcription product. The final volume was adjusted to 20 μL with nuclease-free water. Quantitative reverse transcription polymerase chain reaction (RT-qPCR) was performed as instructed by the Minimum Information for Publication of Quantitative Real-Time PCR Experiment (MIQE) (18). Samples were normalized by the internal control (cyclophilin), and gene expression was quantified by the 2-DDCT method (19). The control group was adjusted for comparison between groups (16,17). All data were expressed as mean ± standard deviation. Intragroup treatments were compared by two-factor analysis of variance (ANOVA) complemented by Tukey's multiple comparison test. The significance level was set at 5% (p < 0.05).

## RESULTS

**Figure 1:**
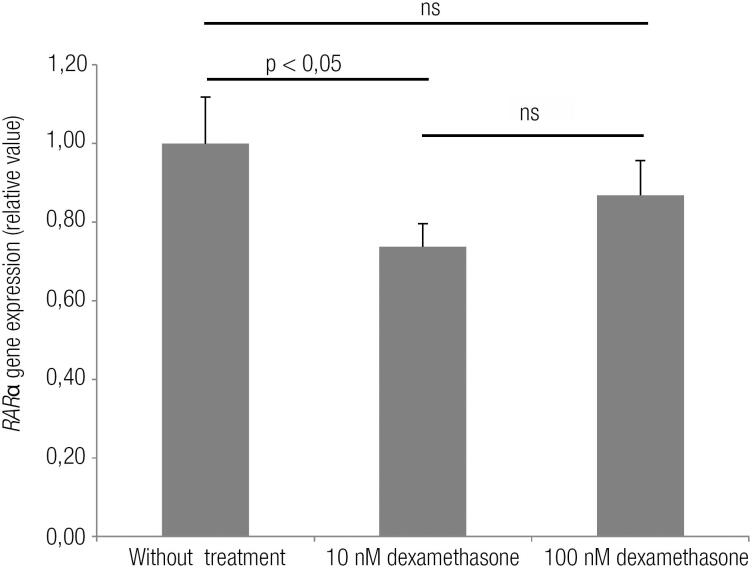
Effect of 10 nM and 100 nM dexamethasone on retinoic acid receptor alpha (*RAR*α) mRNA in orbital fibroblasts from a patient with Graves’ ophthalmopathy. The experiment was performed in triplicate. Data were reported as mean and standard deviation. ANOVA was used in conjunction with Tukey's test (p < 0.05). ns: not significant.

Orbital fibroblasts expressed the *RAR*α gene. Gene expression in cells treated with 10 nM Dex was 24% lower than the control group (p < 0.05). However, gene expression in cells treated with 100 nM Dex was not significantly different from controls or the 10 nM Dex group (Figure 1).

## DISCUSSION

This study confirmed that the OF of GO patients expressed the *RAR*α gene. Lower doses of glucocorticoid reduced *RAR*α gene expression while higher doses did not change it significantly.

RA plays an important role in adipogenesis. Low RA concentrations promote adipocyte differentiation (7), but supraphysiological concentrations inhibit it (6). These findings indicate the importance of studying RA receptors in disorders where adipocytes differentiate and proliferate as a pathophysiological mechanism. Normal human fibroblasts from the lungs and skin express *RAR*α, and its expression is modulated in certain situations, such as types of cell growth conditions, and treatment with ascorbic acid, for example (20,21). Likewise, the OF of patients with GO express *RAR*α, which respond differently, depending on the concomitant medication used (13,15).

An impact of RAR in GO physiopathology could be its role in adipogenesis. Wang and cols. found that mouse adipocytes treated with all-trans-retinoic acid (ATRA) had higher *RAR* expression, *RAR*_γ_ more so than *RAR*α, with consequent decrease of *PPAR*_γ_ expression (14). It is known that *PPAR*_γ_ plays a role in GO development, and recently, we reported that OF from a GO patient treated with small doses of corticosteroid had higher PPARγ gene expression. This could lead to higher differentiation of fibroblasts into adipocytes and speculation that higher glucocorticoid doses would better treat this disease (16).

Yet, in addition to adipogenesis, inflammation also has an important role in GO pathophysiology (1). Thus, vitamin A could act on immunity and inflammation. In autoimmune diseases, RA helps T-cell induction and gene regulation via RAR, which behaves as a transcription factor (8), making RAR essential for preventing and maintaining the tolerance to autoimmune/inflammatory diseases. In light of this, RA treatment could drive distinct pathogenesis evolution by its effects through RAR-modulated pathways in OF, mainly by changing cellular proliferation, differentiation, and apoptosis (13). Indeed, OF from GO patients treated with RA presented morphological alterations, and a decrease in cell growth/proliferation accompanied by the expression of RAR subtypes (13). Moreover, RARα agonist inhibited 36% of the TGF-β1 stimulatory effect on extracellular matrix remodeling and human Tenon fibroblast contractility (22).

Despite this, there is a lack of knowledge regarding the molecular mechanisms of RAR activation in GO. In addition, the OF from GO patients have a hyperresponsive phenotype and consist of several cell subsets, such as Thy1^+^/Thy1^–^, fibrocytes/CD34^+^ (23). Perhaps RAR stimulation in different types of fibroblasts could result in different responses, aimed at adipogenesis or fibrogenesis. Our results show that different doses of glucocorticoid, the main treatment for moderate to severe cases of GO, can lead to a differential *RAR*α expression. This finding suggests that corticotherapy could alter the OF phenotype for *RAR*, and adds another variable to the pathophysiology of OG since it could result in a different response from these receptors to eventual stimuli.

To summarize, in the GO treatment, the appropriate *RAR* expression, modulated by glucocorticoid therapy, would help to reduce adipocyte proliferation and inflammation. As the smaller Dex dose reduced *RAR*α expression, and the higher dose did not affect it, as observed with *PPAR*_γ_ (16), one could speculate that lower glucocorticoid doses would have lesser effects, both anti-inflammatory and in inhibiting adipocyte differentiation. Considering the eventual therapeutic role of retinoids in GO, and the dynamic and sensitive mechanism that modifies RARα mRNA levels in fibroblasts, higher doses of glucocorticoids would be more appropriate if combined with RA.

This study has some limitations, such as using OF from a single patient and not investigating other RA receptors or their protein expression. However, to our knowledge, this was the first study assessing *RAR*α gene expression by the OF from a GO patient treated with different glucocorticoid doses, given that glucocorticoids are currently the main treatment for this ocular disorder.

In conclusion, the orbital fibroblasts from a GO patient expressed the *RAR*α gene, which was not affected by higher, but decreased with lower, glucocorticoid doses. Other studies with more patients are needed to confirm these results.
